# A rare form of Mayer–Rokitansky–Küster–Hauser syndrome associated with ovarian endometrioma: a case report

**DOI:** 10.1093/jscr/rjaa393

**Published:** 2020-09-30

**Authors:** Antoine Naem, Anwar Shamandi, Bashar AL-Kurdy

**Affiliations:** Faculty of Medicine of Damascus University, Damascus, Syria; Faculty of Medicine of Damascus University, Damascus, Syria; University Hospital of Obstetrics and Gynecology, Damascus, Syria

## Abstract

Mayer–Rokitansky–Küster–Hauser syndrome is a congenital malformation that affects the uterus and upper two-thirds of the vagina. Its prevalence is estimated to be 1 in 4500 live births. We present the case of a 19-year-old patient that presented with primary amenorrhea and cyclic abdominal pain. Upon the exploratory laparoscopy, a right rudimentary uterine horn and left unicornuate uterus were found. These two entities were completely separated from each other and from the vaginal vault. In addition, a left ovarian endometrioma was also found. The unicornuate uterus was resected with an intent to resolve the pain. Endometriosis is known to raise the risk of ovarian cancer by 50%. Therefore, a left salpingo-oophorectomy was performed to minimize the risk of ovarian cancer and endometriosis recurrence. In conclusion, ovarian endometriomas should be suspected when obstructive malformations are present with active endometrial remnants. These lesions should be managed appropriately to optimize the postoperative outcomes.

## INTRODUCTION

Mayer–Rokitansky–Küster–Hauser (MRKH) syndrome is defined by the combination of vaginal atresia and different extents of uterine hypoplasia in a normal 46, XX female with normal secondary sexual development [1]. MRKH syndrome results from the interrupted development of the Müllerian ducts during embryogenesis [2]. Its prevalence is estimated to be 1 in 4500 live births [3]. MRKH syndrome could present solely or accompanied by other congenital malformations. On this basis, it was classified into: typical and atypical forms [4]. The typical form refers to the isolated Müllerian agenesis. It is the most common form of the syndrome and accounts for 65% of cases [2]. The atypical form consists of genital malformations accompanied by congenital renal, auditory and skeletal anomalies. This form is less common and referred to as ‘GRES’ syndrome [4]. MRKH syndrome is the second most common cause of amenorrhea [2]. Patients with MRKH syndrome may suffer from cyclic abdominal pain that could be attributed to the presence of active Müllerian remnants [1]. The optimal management remains controversial. However, surgical excision of the rudimentary remnants was considered as the best intervention to relieve the patients’ symptoms [5]. Here, we report a rare form of MRKH syndrome presenting with left ovarian endometrioma. To the best of our knowledge, this malformation has never been reported previously in the literature.

**Figure 1:**
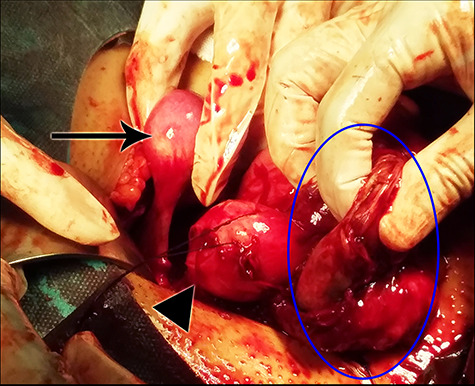
A view from the operation field demonstrating the right rudimentary uterine horn (arrow), the left unicornuate uterus (arrow’s head) and the fibrous wall of the endometrioma (blue circle).

## CASE REPORT

A 19-year-old woman presented to the outpatient clinic at our university hospital complaining of primary amenorrhea. The patient suffered from cyclic lower abdominal pain that occurred monthly. Her medical history was significant for primary hypothyroidism and type 1 diabetes mellitus. The clinical

**Figure 2:**
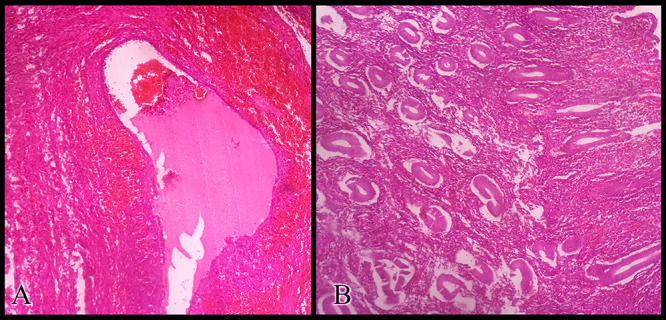
Histopathologic examination of the resected specimens stained with hematoxylin and eosin showing (**A**) the ovarian endometrioma and (**B**) the resected unicornuate uterus with its normal endometrium at the proliferative phase.

examination was unremarkable. The patient’s external genitalia appeared normal. Her breasts’ development, pubic and axillary hair corresponded with Tanner Stage V. Her hormonal profile was completely normal and she had a normal female karyotype (46, XX). Magnetic resonance imaging of her pelvis confirmed the presence of two uterine horns and a blind-ending vagina. The uterine cervix could not be visualized. A left ovarian endometrioma measuring 6 cm was noted. The patient’s urinary, skeletal and respiratory systems were completely normal. The aforementioned findings raised the suspicion of MRKH syndrome. To confirm the diagnosis, an exploratory laparoscopy was performed. A right non-communicating hypoplastic rudimentary uterine horn and a left unicornuate uterus with a hypoplastic cervix were noted. The rudimentary horn and the uterus were completely separated from each other and from the vaginal vault. The left endometrioma was also observed. The diagnosis of a typical MRKH syndrome was confirmed. In order of keeping the vaginal vault intact, the conversion to laparotomy was carried out through a Pfannenstiel incision (Fig. 1). The left unicornuate uterus was resected to relieve the cyclic pain. We were unable to resect the endometrioma completely due to the strong adhesion between its wall and the bowel loops. Therefore, a small part of the cystic wall was kept and a left salpingo-oophorectomy was performed with an intent to minimize the recurrence risk. The histopathologic examination of the resected specimen confirmed the presence of active uterine endometrium and ovarian endometriosis (Fig. 2). The patient’s recovery period was uneventful. Postoperative therapy with cyclic oral contraceptive was administered to prevent the recurrence of endometriosis.

## DISCUSSION

MRKH syndrome is characterized by the absence of the upper two-thirds of the vagina accompanied by various types of uterine anomalies, including the absence of the uterus [1]. It could be isolated or accompanied by other congenital malformations [4]. The main symptom of MRKH syndrome is primary amenorrhea. However, some patients may complain from cyclic lower abdominal pain [1].

The exact cause of the pain remains unknown. One possible reason is the presence of active Müllerian remnants [1]. It is noteworthy that active Müllerian remnants are found in 39% of patients with MRKH syndrome [6]. As the hormonal levels are normal in patients with MRKH syndrome, the Müllerian remnants can undergo cyclic changes and bleed at the end of each menstrual cycle. The menstrual blood will accumulate due to the obstructive anomalies of the uterus and the vagina, which can lead to painful hematometra [7]. Another possible etiology that might exacerbate the pain is endometriosis. One study found that 38% of patients with MRKH syndrome also suffered from endometriosis [1]. Even though the pain’s etiology is controversial, surgical excision of the Müllerian remnants resolved the pain successfully in 50% of patients. Although the pain persisted in only 21% of patients [1], in our case, surgery could not relieve the patient’s pain completely. This could be attributed to the incomplete resection of the endometrioma’s wall. However, combined oral contraceptive administration ameliorated the pain significantly in a previously reported case [7]. In addition, it was also recommended to minimize the recurrence risk of endometriosis postoperatively [8]. This encouraged us to administrate combined oral contraceptive pills to our patient. Moreover, endometriosis raises the risk of ovarian cancer by 50%. Salpingo-oophorectomy was found to be the most feasible risk reduction intervention [8].

The coexistence of endometriosis and MRKH syndrome adds more controversies to the endometriosis pathogenesis theories. Some authors indicated that endometriosis may present in patients with MRKH syndrome even when the active Müllerian remnants are absent [9]. However, a recent review of the literature showed that a considerable amount of cases failed to detect the active endometrium radiologically even when it was found microscopically. Moreover, active Müllerian remnants were found in every patient with MRKH syndrome that suffered from endometriosis [10]. These findings support the retrograde menstruation theory.

## CONCLUSIONS

MRKH syndrome can have various forms of Müllerian agenesis. Ovarian endometriomas should be suspected when obstructive malformations and active endometrial remnants are present. Endometriomas should be considered as a potential contributor to the patient’s symptoms. Appropriate management of endometriosis is crucial for optimizing the postoperative outcomes as well as to reduce the risk of ovarian cancer and endometriosis recurrence.
